# Risk factors for eczema in infants born in Cuba: a population-based cross-sectional study

**DOI:** 10.1186/1471-5945-14-6

**Published:** 2014-03-25

**Authors:** Ramón Suárez-Medina, Silvia Josefina Venero-Fernández, Esperanza de la Mora-Faife, Gladys García-García, Ileana del Valle-Infante, Liem Gómez-Marrero, Dania Fabré-Ortiz, Hermes Fundora-Hernández, Andrea Venn, John Britton, Andrew W Fogarty

**Affiliations:** 1 Instituto Nacional de Higiene, Epidemiología y Microbiología, Infanta No 1158 e/ Llinásy Clavel, Código Postal 10300 La Habana, Cuba; 2 Hospital Pediátrico Docente “Juan Manuel Márquez”, La Habana, Cuba; 3 Nottingham Biomedical Research Unit, Division of Epidemiology and Public Health, University of Nottingham, Clinical Sciences Building, City Hospital, NG5 1 PB Nottingham, UK

**Keywords:** Eczema, Infants, Risk factor, Cuba, Paracetamol

## Abstract

**Background:**

There is a concern that allergic disease in childhood is higher than expected in Cuba. The aim of this study was to determine the risk factors for eczema of infants aged 12–15 months living in Havana.

**Methods:**

We used a cross-sectional epidemiological study design. Data on eczema symptoms and a wide range of lifestyle factors were collected by researcher administered questionnaires.

**Results:**

Data were collected on 1956 children (96% response rate), of whom 672 (34%) were reported as having had eczema. Independent risk factors for eczema included young maternal age (adjusted odds ratio (OR) 0.98 per additional year of age; 95% confidence interval (CI) 0.97-0.99), child’s weight (OR 1.13 per additional kg; 95% CI: 1.03-1.25), insect sting allergy (OR 2.11; 95% CI: 1.33-3.35), rodents in the home (OR 1.39; 95% CI: 1.10-1.76), attendance at childcare facilities (OR 1.34: 95% CI: 1.05-1.70) and self-reported mould in the home (OR 1.23; 95% CI: 1.07-1.41). Infant exposure to paracetamol was associated with an increased risk of eczema even after adjustment for wheeze (OR 1.22; 95% CI: 1.03-1.46).

**Conclusion:**

Despite a very different culture and environment, the consistency of these findings with those from more economically developed countries suggests potential causal associations. The association with paracetamol, even after adjustment for wheeze, suggests that intervention studies are required in young infants, to ascertain if this commonly used anti-pyretic medication increases allergic disease.

## Background

Allergic disease is increasing globally [1], particularly in countries where it was previously relatively rare [2], and is most common in the more affluent, urbanised and economically developed countries. However the aetiological factors responsible remain unknown. It is likely that much of the increase in allergic disease is the result of composite exposures, probably starting *in utero*, which cause disease in susceptible individuals [3]. Hence studies from the early years of childhood are important in identifying risk factors for subsequent development of common allergic diseases such as eczema, which will aid our understanding of the environmental exposures that result in allergic phenotypes.

One avoidable risk factor of particular interest in the aetiology of allergic disease is use of paracetamol, which has been shown to be associated with eczema in both children and adults [4-7]. However, these associations are potentially confounded by the use of paracetamol to treat symptoms of respiratory infection, which are a common manifestation of childhood asthma. It is therefore important to adjust for this confounding effect, to determine whether paracetamol use and eczema are independently linked.

Cuba has a health care system that delivers infant mortality rates comparable to much richer countries [8], and deals well with common conditions that might be misclassified as eczema. However, as a consequence of the economic embargo on the island imposed by the USA, societal development has been constrained. The combination of good health infrastructure with limited economic growth in recent decades has resulted in a unique environment that provides an ideal setting where risk factors for disease can be studied utilising experienced staff based in the network of policlinics that provide healthcare to the whole population. As the pathogenetic pathways that result in disease are likely to be consistent across human populations, risk factors for allergic disease from Cuba that are consistent with those identified in more affluent countries are more likely to be real and not simply artefacts due to confounding.

In the recent phase three of the International Study of Asthma and Allergies in Childhood (ISAAC), Cuba had one of the highest prevalences of eczema among the countries studied from Latin America [9]. This study concluded that ‘environmental risk factors must be evaluated in order to identify potential causes for the differences observed’. To address these issues, we have used a cross-sectional study design to explore the risk factors for eczema in the first year of life for infants born in Havana, Cuba.

## Methods

### Study population

All children aged between 12–15 months who were living in Havana, Cuba between March 2010 and March 2011 and who attended one of randomly selected 17 policlinics, nested in four municipalities in Havana, Cuba were eligible to be selected to participate in the study (Arroyo Naranjo, Cerro, Habana del Este, La Lisa). Selection of the study population has been described elsewhere [10]. The study protocol was approved by National Institute of Hygiene, Epidemiology and Microbiology, the local Havana Scientific Committee and also by the University of Nottingham Medical School ethics committee. All parents/guardians provided written informed consent for the infants to participate in the study.

### Data collection

The baseline data collection consisted of an interviewer administered questionnaire (on-line Additional file 1) that collated the responses from the parent/carer about demographics of the child and mother, and a wide range of prenatal and postnatal exposures of the child including their living environment, the medical history of the family, exposure to medications such as paracetamol, aspirin and antibiotics, and exposure to environmental tobacco smoke. Data on the height and weight at both the time of birth and the interview were also collected. Allergic disease outcomes were collected using the Spanish translation of the ISAAC questionnaire [9,11] which was piloted in the local community. The outcome of interest, eczema, was based on the following question: ‘Does your infant have or has had an itchy rash in any of the following places: the folds of the elbows, behind the knees, in front of the ankles, under the buttocks, or around the neck, ears or eyes during the first year of life?’ (¿Su bebé tiene o ha tenido erupción con picazón en los siguientes lugares: sitio de flexión del brazo, atrás de las rodillas, en las muñecas, debajo de las nalgas o alrededor del cuello, orejas y ojos durante el primer año de vida?). Data on insect sting allergy were collected using the question “Has your baby been diagnosed with insect sting allergy in the first year of life”.

### Data analysis

All statistical analyses were carried out in Statav12 (StataCorp, Texas, USA) using the survey commands to allow for the clustered survey design. Univariate analyses were initially performed using logistic regression. Variables that were statistically significant in univariate analysis (p ≤ 0.05) were then entered into a mutually adjusted multivariable mode and a step-wise modelling procedure followed to obtain a final model of only statistically significant (p ≤ 0.05) variables. Gender was considered as *a priori* confounder. Associations of the outcome with exposures that were continuous variables were checked for linearity using likelihood ratio tests. The presence of self-reported wheeze was considered in the univariate analyses but omitted from the final model, as this is another manifestation of allergic disease, which would result in overfitting.

### Sample size

We aimed to collect data on 2000 children. In the first year of life eczema is a better measure of allergic disease than wheeze and we have used this for the primary power calculation. We anticipate that we will have over 95% power (Epi-info, StatCalc) to detect an increase of the odds ratio of eczema of 1.9 for those who receive any paracetamol in the first year of life compared to those who do not receive paracetamol conservatively assuming a baseline prevalence of eczema of 20% and that 10% of the children in the cohort will receive paracetamol at some point in the first year of life.

## Results

Of the 2032 infants who were eligible for inclusion in the study, 1956 provided data giving a 96% response rate. The characteristics of the study participants are presented in Table 1 and the distribution of the risk factors of interest in Table 2. 672 (34%) infants were reported as having had eczema symptoms in the first year of life. This was strongly associated with other allergic disease outcomes measured, including reported wheeze (odds ratio (OR) 2.35, 95% confidence intervals (CI) 1.93, 2.85), asthma diagnosis (OR 1.97, 95% CI: 1.45-2.67) and allergic rhinitis (OR 2.48, 95% CI: 2.00, 3.08).

**Table 1:** Characteristics of study participants

**Variable**	**Definition of category**	**Number (%) unless stated otherwise**	**Prevalence of eczema (%)**	**Univariate OR for eczema (95% CI) using survey commands**
Mean age months, (sd)		13.1 (1.1)	n/a	1.05 (0.91-1.20) per month
Skin colour (%)	White	916 (47)	301 (33)	1
	Mixed	798 (41)	282 (35)	1.12 (0.73-1.70)
	Black	242 (12)	89 (37)	1.19 (0.82-1.73)
Gender (%)	Female	939 (48)	301 (32)	1
	Male	1017 (52)	371 (36)	1.22 (0.90-1.64)
Municipality (%)	Habana del	642 (33)	240 (37)	1
	Este			
	Cerro	374 (19)	81 (22)	0.46 (0.39-0.56)
	La Lisa	282 (14)	109 (39)	1.06 (0.84-1.32)
	Arroyo Naranjo	658 (34)	242 (37)	0.97 (0.80-1.19) p = 0.06
Highest educational status of mother (%)	Primary	17 (1)	9 (53)	1
	Secondary	431 (22)	159 (37)	0.52 (0.15-1.83)
	Pre-university	1157 (59)	382 (33)	0.44 (0.10-1.88)
	University	351 (18)	122 (35)	0.47 (0.17-1.31) p = 0.59 p_(trend)_ = 0.18
Mother with paid work (%)	No	780 (40)	264 (34)	1
	Yes	1176 (60)	408 (35)	1.04 (0.72-1.51)
Household income pesos, (%)	>3000	35 (2)	9 (26)	1
	2000-3000	48 (2)	21 (44)	2.25 (0.69-7.34)
	1001-1999	207 (11)	76 (37)	1.68 (0.41-6.81)
	500-1000	955 (49)	335 (35)	1.56 (0.38-6.41)
	<500	711 (36)	231 (32)	1.39 (0.42-4.55) p = 0.85 p_(trend)_ = 0.44
Any siblings (%)	No	818 (42)	297 (36)	1
	Yes	1138 (58)	375 (33)	0.86 (0.63-1.18)
Mean age of mother at birth in years (sd)	-	26.7 (6.2)	n/a	0.98 (0.97-0.99) per year
Reported eczema	No	1284 (66)	n/a	
	Yes	672 (34)		

**Table 2:** Univariate analysis of exposures and risk of eczema since birth

**Variable**	**Definition of category**	**Number (%) unless stated otherwise**	**Prevalence of eczema (%)**	**OR for eczema using survey command (95% CI)**
Mother used paracetamol in pregnancy (%)	No	1809 (92)	626 (35)	1
	Yes	147 (8)	46 (31)	0.86 (0.43-1.73)
Mother used aspirin in pregnancy (%)	Never	1778 (91)	596 (34)	1
	Yes	178 (9)	76 (43)	1.48 (0.72-3.05)
Infant’s mean birth weight, kg, (sd)		3.34 (0.53)	n/a	0.98 (0.84-1.13) per kg
Infant’s mean height at birth cm (sd)*		50.28 (2.38)	n/a	1.02 (0.99-1.06) per cm
Infant’s mean weight, kg, (sd)		10.52 (1.56)	n/a	1.14 (1.04-1.26) per kg
Infant’s mean height, cm (sd)		74.67 (3.72)	n/a	1.02 (1.00-1.03) per cm
Caesarean birth (%)	No	1163 (59)	394 (34)	1
	Yes	793 (41)	278 (35)	1.05 (0.73-1.52)
Respiratory distress at birth (%)	No	1836 (94%)	620 (34)	1
	Yes	120 (6%)	52 (43)	1.50 (1.04-2.16)
Breastfeeding ≥4 months	No	890 (45)	337 (38)	1
	Yes	1066 (55)	335 (31)	0.75 (0.56-1.01)
Family history of asthma	No	917 (47)	267 (29)	1
	Yes	1039 (53)	405 (39)	1.56 (1.32-1.83)
Insect sting allergy (%)	No	933 (48)	229 (25)	1
	Yes	1022 (52)	443 (43)	2.35 (1.56-3.56)
	Missing	1		
Paracetamol use by infant including kogrip (%)	No	1486 (76)	486 (33)	1
	Yes	470 (24)	186 (40)	1.35 (1.12-1.62)
Antibiotic use by infant	No	1255 (64)	363 (29)	1
	Yes	696 (36)	307 (44)	1.94 (1.40, 2.68)
State of home (%)	Good	1368 (70)	477 (35)	1
	Regular	463 (24)	149 (32)	0.87 (0.77-1.01)
	Poor	125 (6)	46 (37)	1.09 (0.40-2.98) p = 0.13 p_(trend)_ = 0.84
No. of rooms in house (excluding bathroom and kitchen)	1	246 (13)	91 (37)	1
	2	540 (28)	167 (31)	0.76 (0.38-1.54)
	≥3	1170 (60)	414 (35)	0.93 (0.44-1.99) p = 0.35 p_(trend)_ = 0.83
Ventilation of home (%)	Good	1535 (78)	524 (34)	1
	Regular	307 (16)	102 (33)	0.96 (0.72-1.28)
	Poor	114 (6)	46 (40)	1.31 (0.70-2.44) p = 0.62 p_(trend)_ = 0.31
Presence of ornamental plants (%)	No	1405 (72)	484 (34)	1
	Yes	551 (28)	188 (34)	0.99 (0.77-1.26)
Presence of bathroom in home (%)	No	255 (13)	96 (38)	1
	Yes	1701 (87)	576 (34)	0.85 (0.59-1.22)
Mould in home (%)	No	1385 (71)	443 (32)	1
	Yes	571 (29)	229 (40)	1.42 (1.19-1.70)
Child sleeps in cooking area	No	1774 (91)	592 (33)	1
	Yes	182 (9)	80 (44)	1.57 (1.17-2.10)
Cook with gas (%)	No	133 (7)	64 (48)	1
	Yes	1823 (93)	608 (33)	0.54 (0.32-0.90)
Cook with electricity (%)	No	1507 (77)	482 (32)	1
	Yes	449 (23)	190 (449)	1.56 (0.99-2.45)
Mosquito nets in home (%)	No	728 (37)	240 (33)	1
	Yes	1228 (63)	432 (35)	1.10 (0.83-1.47)
Air conditioning (%)	No	1619 (83)	565 (35)	1
	Yes	337 (17)	107 (32)	0.87 (0.72-1.05)
Infant’s room walls painted before birth	No	953 (49)	321 (34)	1
	Yes	1003 (51)	351 (35)	1.06 (0.85-1.32)
Infant’s room walls painted after birth	No	1713 (88)	580 (34)	1
	Yes	243 (12)	92 (38)	1.19 (0.66-2.16)
Infant’s mattress	Used	1062 (54)	374 (35)	1
	New	894 (46)	298 (33)	0.92 (0.67-1.26)
Infant sleeps alone	No (not alone)	829 (42)	297 (36)	1
	Yes (alone)	1127 (58)	375 (33)	0.89 (0.62-1.30)
Daily use of soap	No	69 (4)	26 (38)	1
	Yes	1887 (96)	646 (34)	0.86 (0.33-2.22)
Use of shampoo	No	615 (31)	209 (34)	1
	Yes	1341 (69)	463 (35)	1.02 (0.69-1.52)
No. of people in household	2	237 (12)	75 (32)	1
	3	485 (25)	170 (35)	1.17 (0.54-2.49)
	4	551 (28)	178 (32)	1.03 (0.50-2.14)
	5	332 (17)	116 (35)	1.16 (0.74-1.83)
	≥6	351 (18)	133 (38)	1.32 (0.63-2.74) P = 0.80 p_(trend)_ = 0.20
Eats vegetables	No	350 (18)	115 (33)	1
	Yes	1606 (82)	557 (35)	1.08 (0.75-1.57)
Eats fruit	No	184 (9)	65 (35)	1
	Yes	1772 (91)	607 (34)	0.95 (0.49-1.85)
Maternal smoking during pregnancy	No	1779 (91)	609 (34)	1
	Yes	177 (9)	63 (36)	1.06 (0.67-1.67)
Mother currently smokes	No	1557 (80)	513 (33)	1
	Yes	399 (20)	159 (40)	1.35 (0.92-1.97)
Father currently smokes	No	1309 (67)	434 (33)	1
	Yes	647 (33)	238 (37)	1.17 (0.83-1.65)
Number of smokers in home	0	952 (49)	299 (31)	1
	1	494 (25)	176 (36)	1.21 (0.90-1.62)
	2	340 (17)	131 (39)	1.37 (0.86-2.17)
	≥3	170 (9)	66 (39)	1.39 (0.89-2.15) P = 0.31 p_(trend)_ = 0.03
Any grandparents smoke	No	832 (43)	274 (33)	1
	Yes	1124 (57)	398 (35)	1.12 (0.83-1.50)
Pets in home at time of birth	No	1259 (64)	417 (33)	1
	Yes	697 (36)	255 (37)	1.16 (0.70-1.93)
Pets in home now	No	1239 (63)	419 (34)	1
	Yes	717 (37)	253 (35)	1.07 (0.74-1.52)
Rodents in home	No	1643 (84)	534 (32)	1
	Yes	313 (16)	138 (44)	1.64 (1.33-2.02)
Cockroaches in home	No	1432 (73)	470 (33)	1
	Yes	524 (27)	202 (39)	1.28 (0.77-2.14)
Air pollution near home	No	1407 (72)	476 (34)	1
	Yes	549 (28)	196 (36)	1.09 (0.57-2.08)
Child attended daycare/nursery	No	1685 (86)	559 (33)	1
	Yes	271 (14)	113 (42)	1.44 (1.14-1.83)
Any parasites**	No	686 (96)	262 (38)	1
	Yes	26 (4)	9 (35)	0.86 (0.23-3.21)
Any wheeze	No	1084 (55)	280 (26)	1
	Yes	872 (45)	392 (45)	2.34 (1.56-3.53)

*5 missing values.

**1244 infants were unable to provide a faeces sample for analysis.

The univariate associations with eczema are shown in Table 1 and Table 2. In the multivariate analysis those variables identified as statistically significant independent risk factors for eczema were young maternal age, increasing child’s weight, family history of asthma, insect sting allergy, attendance at childcare facilities, use of antibiotics, use of preparations that contain paracetamol, mould in the home and rodents in the home (Table 3).

**Table 3:** Multivariate analysis of exposures and risk of eczema in first year of life

**Variable**	**Definition of category**	**Number**	**Adjusted OR for any eczema (95**% **CI)**
Gender	Female		1
	Male		1.09 (0.76-1.57)
Infant’s mean current weight, per kg*	-	10.52 (1.56)	1.13 (1.03-1.25)
Family history of asthma	No	917 (47)	1
	Yes	1039 (53)	1.34 (1.04-1.73)
Insect sting allergy (%)	No	933 (48)	1
	Yes	1022 (52)	2.11 (1.33-3.35)
Use of paracetamol	No	1486 (24)	1
	Yes	470 (76)	1.22 (1.02-1.46)
Use of antibiotics	No		1
	Yes		1.61 (1.10-2.34)
Age of mother at birth (per year*)	-	26.7 (6.2)	0.98 (0.97-0.99)
Rodents in home	No	1643 (84)	1
	Yes	313 (16)	1.39 (1.10-1.76)
Mould in home (%)	No	1385 (71)	1
	Yes	571 (29)	1.23 (1.07-1.41)
Attended daycare/nursery	No	1685 (86)	1
	Yes	271 (14)	1.34 (1.05-1.70)

1937 individuals provided complete data for analysis.

*linear association.

The effect of maternal age was such that the risk of eczema decreased by an estimated 2% for every additional year of age of the mother. Similarly, a linear effect of child’s weight at the time of the interview was seen, with an estimated 13% increase in eczema risk per additional kg in child’s weight; birthweight however was not associated with eczema. To investigate whether the observed relationship between child’s paracetamol use and eczema may be confounded by indication (children with eczema more likely to experience respiratory symptoms which may be treated with paracetamol), we adjusted for symptoms of wheeze and this made little change to the association with paracetamol OR (1.22; 95% CI: 1.03, 1.46)). The effect of antibiotics however did weaken when adjusted for wheeze in the first year of life, and was no longer statistically significant (OR 1.24; 95% CI 0.91, 1.69).

## Discussion

Cuba is unique, having a high quality health infrastructure despite a weak economy that is limited by political circumstance, which facilitates the delivery of high quality observational epidemiological studies in a relatively poor country. To our knowledge, this is the first large epidemiological study to explore the risk factors for eczema in infants in Cuba in the 21^st^ century, a country that is known to have a very high, unexplained prevalence of symptoms of eczema in children [9]. We have identified that insect sting allergy, family history of asthma, presence of rodents or mould in the house, day-care attendance, a heavier current weight, having a younger mother as independent risk factors for reported symptoms of eczema in Havana. The association with paracetamol is particularly interesting as it persisted even after adjustment for wheeze, suggesting that this was not simply confounding by association with respiratory infections. It is also of pragmatic importance since exposure to paracetamol in this population, as in developed countries, was common.

Our data have a number of strengths that give us confidence in the integrity of these observed associations. In particular, the relatively large sample of almost 2000 infants with a response rate of 96% is excellent, and a consequence of nesting the data collection within the policlinics minimising inconvenience to the families and staff involved. This reduces the risk of response bias and also permits the associations observed to be generalised to other similar populations. The study questionnaire was developed by local epidemiologists who have much experience of work with these populations and hence allowed consideration of local factors that may be specific to these localities in the analysis. The data were collected by highly trained health professionals who knew the participants, thus enhancing the quality of the information collected.

There are some limitations of our study design, which are unavoidable. Cross-sectional study designs do not allow causality to be attributed, and only associations to be identified. However, the nature of the hypothesis we were testing does not make reverse causality a likely explanation for the relationships that were observed. We were unable to do any detailed measurements when the infants were *in utero*, although we were able to collect data on important exposures related to pregnancy such as maternal smoking in pregnancy, weight and height at birth, the mode of delivery and the duration of breastfeeding. The use of the ISAAC questionnaire definition of eczema allows comparison of our data with other similar studies [11], but it is possible that this practical epidemiological approach to defining the outcome measure may have a degree of imprecision as other causes of itchy rashes in addition of eczema may be included in the outcome measure. We are unable to exclude the possibility of recall bias and residual confounding influencing our data and the associations reported. Finally, we have tested a number of exposures and hence are unable to exclude the possibility that some of the associations we observed are the consequence of chance.

There have been no comparable studies of eczema in infants in Cuba, and so it is difficult to directly compare our data with other study datasets. The third phase of the ISAAC study had a limb in Havana and reported a prevalence of having ever had a eczematous rash of 26%, one of the higher prevalences in Latin America in children aged six to seven years [9] with the data collected in the latter half of the first decade of the 21^st^ century. A rash was reported to have first occurred below the age of two years in 15% of these children, compared to values of less than 1% in centres in Mexico. This is compared to our prevalence of 34% of children having symptoms of eczema in the first year of life in 2010. Obviously these two prevalence estimates are not directly comparable due to differences in definitions and recall bias, but it is possible that eczema prevalence has increased in young infants living in Havana over the first decade of the 21^st^ century.

This is the first study to report an association between paracetamol exposure and eczema after adjustment for symptoms of wheeze in young infants. The only other dataset that studied the impact of paracetamol exposure on incident eczema in 780 children aged between 1 and 3 years living in Ethiopia that adjusted for symptoms of respiratory infections did not observe a similar association [12], although the different ages prevent direct comparison with our population, while the baseline analysis from the same study again did not observe an association with eczema with use of paracetamol [13]. Other studies have reported that paracetamol is a risk factor for eczema using analyses in older children from international datasets [4,6,7,14,15], and also data from a different population of Ethiopian children and adults from the country reported an odds ratio of 1.9 for risk of self-reported eczema in those taking more than three tablets of paracetamol in the past month compared to those who took none [5]. However, although plausible biological mechanisms exist that may possibly explain how paracetamol exposure in early life can result in allergic disease [15], it should be stressed that the role of paracetamol on the aetiology of allergic disease is contentious and the likelihood is that a randomised controlled trial will be required to resolve these issues.

Many of the other risk factors for symptoms of eczema in our dataset have been previously reported and while not novel, these give us confidence in the integrity of the data collected and associations observed. Hence, it is well known that a family history of asthma is a risk factor for allergic diseases such as eczema in children [16] while the presence of mould in the home is also well recognised to be associated with allergic disease [17]. We observed a strong association between a diagnosis of insect sting allergy in the first year of life with eczema symptoms that appears plausible in the light of other studies. One study population from Israel of 13 to 14 year old schoolchildren reported an increased in both rate and severity of allergic reactions to insect stings among those with atopic diseases compared to those who did not have atopic disease [18]. Similarly, a population-based study from the USA reported that people with atopy experienced anaphylaxis, an extreme form of allergic reaction more frequently than people without atopy [19].

To our knowledge, the inverse association between maternal age and risk of eczema has not been reported previously [20-23]. Maternal age is closely related to birth order as older mothers have more children, and this is considered to be a risk factor for allergic disease, with individuals with more elder siblings having less hayfever and eczema [24]. Cord blood IgE is a risk factor for development of atopic disease in childhood, and Scirica reported that older mothers have lower levels of cord blood IgE using data from 874 children from the USA [25], an observation that is compatible with our data. The association between presence of rats in the home and eczema is difficult to explain using the existing literature but we speculate that this could be a surrogate marker for living conditions or other exposures that we were unable to measure.

## Conclusion

In conclusion, we report that in infants born in Havana, Cuba, insect sting allergy, family history of asthma, presence of rodents or mould in the house, daycare attendance, a heavier current weight, use of paracetamol, and having a younger mother are all independent risk factors for reported symptoms of eczema. The delivery of high quality observational epidemiological studies in a developing country is challenging for many reasons, but good data from these environments are important as associations that are observed consistently across a range of societies are more likely to be causal and not simply a consequence of confounding, possibly by affluence. Cuba is a unique developing country with excellent public health provision and diagnostic ascertainment, and the consistency of these findings with those from a range of more economically developed countries suggests that the risk factors identified in our study may be important in the development of eczema. The association with paracetamol, even after adjustment for wheeze, suggests that intervention studies are required in young infants, to ascertain if this commonly used anti-pyretic medication increases allergic disease.

## Abbreviations

ISAAC: International study of asthma and allergies in childhood; HINASIC: Historia Natural de la Sibilancia en Cuba/National History of Wheezing in Cuba.

## Competing interests

There are no competing interests among any of the authors between the reporting of the study findings and financial or non-financial interests that may bias the report.

## Authors’ contributions

The 8 Cuban authors (SF, RS, EM, GG, IV, LG, DF, HF) came up with the original concept of the study and the study design. AF, AV and JB helped with the study design and obtaining funding. The data were collected by the 8 Cuban authors (SF, RS, EM, GG, IV, LG, DF, HF) and analysed by RS, AF and AV. All authors contributed to the final drafting and approval of the manuscript.

## Pre-publication history

The pre-publication history for this paper can be accessed here:

http://www.biomedcentral.com/1471-5945/14/6/prepub

## Supplementary Material

Additional file 1 Study Questionnaire. Click here for file

## References

[B1] Asher M Montefort S Bjorksten B Lai C Strachan D Weiland S Williams H and the ISAAC phase three study group Worldwide trends in the prevalence of symptoms of asthma, allergic rhinoconjunctivitis, and eczema in childhood: ISAAC phases one and three repeat multicountry cross-sectional surveys Lancet 2006 368 733 743 10.1016/S0140-6736(06)69283-0 16935684

[B2] Williams H Stewart A von Mutius E Cookson W Anderson R and the ISAAC phase one and phase three study groups Is eczema really on the increase worldwide? J Allergy Clin Immunol 2008 121 947 954 10.1016/j.jaci.2007.11.004 18155278

[B3] Williams H Robertson C Stewart A Ait-Khaled N Anabwani G Anderson R Asher I Beasley R Bjorksten B Burr M Clayton T Crane J Ellwood P Keil U Mallol J Martinez F Mitchell E Montefort S Pearce N Shah J Sibbald B Strachan D von Mutius E Weiland S Worldwide variations in the prevalence of symptoms of atopic eczema in the international study of asthma and allergies in childhood J Allergy Clin Immunol 1999 103 125 138 10.1016/S0091-6749(99)70536-1 9893196

[B4] Cohet C Cheng S MacDonald C Baker M Foliaki S Huntington N Douwes J Pearce N Infections, medication use, and the prevalence of symptoms of asthma, rhinitis, and eczema in childhood J Epidemiol Community Health 2004 58 852 857 10.1136/jech.2003.019182 15365112 PMC1763349

[B5] Davey G Berhane Y Duncan P Aref-Adib G Britton J Venn A Use of acetaminophen and the risk of self-reported allergic symptoms and skin sensitisation in Butajira, Ethiopia J Allergy Clin Immunol 2005 116 863 868 10.1016/j.jaci.2005.05.045 16210062

[B6] Beasley R Clayton T Crane J Von Mutius E Lai C Monetfort S Stewart A Monetfort S Stewart A and ISAAC phase three study group Association between paracetamol use in infancy and childhood, and risk of asthma, rhinoconjunctivitis, and eczema in children aged 6–7 years: analysis from phase three of the ISAAC progranmme Lancet 2008 372 1039 1048 10.1016/S0140-6736(08)61445-2 18805332

[B7] Beasley R Clayton T Crane J Lai C Montefort S von Mutius E Stewart A and ISAAC phase three study group Acetaminophen use and risk of asthma, rhinoconjunctivitis, and eczema in adolescents Am J Respir Crit Care Med 2011 183 171 178 10.1164/rccm.201005-0757OC 20709817

[B8] Cooper R Kennelly J Ordunez-Garcia P Health in Cuba Int J Epidemiol 2006 35 817 824 10.1093/ije/dyl175 16931525

[B9] Sole D Mallol J Wandalsen G Aguirre V Group LAIPS Prevalence of symptoms of eczema in Latin America: results of the international study of asthma and allergies in childhood (ISAAC) phase 3 J Investig Allergol Clin Immunol 2010 20 311 323 20815309

[B10] Venero-Fernandez S Suarez Medina R Mora Faife E García García G del Valle II Gomez-Marrero L Abreu-Suarez G Gonzalez-Valdez J Fabro-Ortiz D Fundora-Hernadez H Venn A Britton J Fogarty AW Risk factors for wheezing in infants born in Cuba Q J Med 2013 106 1023 1029 10.1093/qjmed/hct143 PMC3808789 23824939

[B11] Wordemann M Polman K Diaz R Heredia L Madurga A-M Sague K Gryseels B Gordea M The challenge of diagnosing atopic diseases: outcomes in Cuban children depend on definition and methodology Allergy 2006 61 1125 1131 10.1111/j.1398-9995.2006.01129.x 16918517

[B12] Amberbir A Medhin G Alem A Britton J Davey G Venn A The role of acetaminophen and geohelminth infection on the incidence of wheeze and eczema Am J Respir Crit Care Med 2011 183 165 170 10.1164/rccm.201006-0989OC 20935107 PMC3040388

[B13] Belyhun Y Amberbir A Medhin G Erko B Hanlon C Venn A Britton J Davey G Prevalence of risk factors of wheeze and eczema in 1-year-old children: the Butajira birth cohort, Ethiopia Clin Exp Allergy 2010 40 619 626 10.1111/j.1365-2222.2010.03479.x 20447078

[B14] Newson R Shaheen S Chinn S Burney P Paracetamol sales and atopic disease in children and adults: an ecological analysis Eur Respir J 2000 16 817 823 10.1183/09031936.00.16581700 11153577

[B15] Holgate S The acetaminophen enigma in asthma Am J Respir Crit Care Med 2011 183 147 151 10.1164/rccm.201007-1135ED 21242591

[B16] Tattersfield A Knox A Britton J Hall I Asthma Lancet 2002 360 1313 1322 10.1016/S0140-6736(02)11312-2 12414223

[B17] Mendell M Mirer A Cheung K Tong M Douwes J Respiratory and allergic health effects of dampness, mold, and dampness-related agents: a review of the epidemiological evidence Environ Health Perspect 2011 119 748 756 10.1289/ehp.1002410 21269928 PMC3114807

[B18] Graif Y Romano-Zelekha O Livne I Green M Shohat T Increased rate and greater severity of allergic reactions to insect sting among schoolchildren with atopic diseases Pediatr Allergy Immunol 2009 20 757 762 10.1111/j.1399-3038.2009.00863.x 19397756

[B19] Yocum M Butterfield J Klein J Volcheck G Schroeder D Silverstein M Epidemiology of anaphyaxis in Olmsted country: a population-based study J Allergy Clin Immunol 1999 104 452 456 10.1016/S0091-6749(99)70392-1 10452770

[B20] Martinez F Wright A Holberg C Morgan W Taussig L Maternal age as a risk factor for wheezing lower respiratory illnesses in the first year of life Am J Epidemiol 1992 136 1258 1268 1476148 10.1093/oxfordjournals.aje.a116434

[B21] Taylor B Wadsworth J Golding J Butler N Breast feeding, eczema, asthma, and hayfever J Epidemiol Community Health 1983 37 95 99 10.1136/jech.37.2.95 6886591 PMC1052269

[B22] Sariachvili M Droste J Dom S Wieringa M Hagendorens M Stevens W van Sprundel M Desager K Weyler J Early exposure to solid foods and the development of eczema in children up to 4 years of age Pediatr Allergy Immunol 2010 21 74 81 10.1111/j.1399-3038.2009.00899.x 19573205

[B23] Butland B Strachan D Lewis S Bynner J Butler N Britton J Investigation into the increase in hay fever and eczema at age 16 observed between the 1958 and 1970 British birth cohorts Brit Med J 1997 315 717 721 10.1136/bmj.315.7110.717 9314757 PMC2127494

[B24] Strachan D Family size, infection and atopy: the first decade of the “hygiene hypothesis” Thorax 2000 55 S2 S10 10.1136/thorax.55.suppl_1.S2 10943631 PMC1765943

[B25] Scirirca C Gold D Ryan L Abulkerim H Celedon J Platts-Mills T Naccara L Weiss S Litonjua A Predictors of cord blood IgE levels in children at risk of asthma and atopy J Allergy Clin Immunol 2007 119 81 88 10.1016/j.jaci.2006.09.002 17208588

