# Supplementary material for: Risk factors for eczema in infants born in Cuba: a population-based cross-sectional study
Source: BMC Dermatol. 2014 Mar 25;14:6. doi: 10.1186/1471-5945-14-6 (PMC3987161; doi:10.1186/1471-5945-14-6)
Supplement: Additional file 1 — Study Questionnaire. [file 1471-5945-14-6-S1.docx]

**ANEXX 3: QUESTIONNAIRE FOR RESPIRATORY SYMPTOMS DURING FIRST YEAR OF LIFE AND RELATED FACTORS.**

| **Questionnaire number:** |  |  |  |  |  |
| --- | --- | --- | --- | --- | --- |

Dear Mom: As part of the research on the knowledge of respiratory diseases of children in our country, we ask you please answer the following questionnaire. Please do not leave boxes blank.

Thank you very much for your valuable cooperation. Information given us will be useful. If you have questions about this survey can clarify directly with us at: Department of Epidemiology, National Institute of Hygiene, Epidemiology and Microbiology Cuba, Address: Infanta # 1158 e/ Clavel y Llinás, Centro Habana. Office phone: 878 8479 or email: [silviavf@inhem.sld.cu](mailto:silviavf@inhem.sld.cu)

**General information**

Person who give the data (choose one):

O 1. Mother O 2. Father O 3. Other_____________

**Demographic and socio- economic data**

1. Child full name: _______________________________________

| 1. Number of National Identity: |  |  |  |  |  |  |  |  |  |  |  |
| --- | --- | --- | --- | --- | --- | --- | --- | --- | --- | --- | --- |

1. Address: ________________________________________________________

________________________________________________________

1. Municipality (choose one):

O 1. Cerro O 2. Habana del Este

O 3. La Lisa O 4. Arroyo Naranjo

1. Policlinic: _______________
2. Collecting date: ____/____/______ Día / mes/ año
3. Contact phone: ______________________
4. Date of birth: ____/____/______ Día / mes/ año
5. Age: _________ (completed months)
6. Sex: O 1. Female O 2. Male
7. Please mark education level attained by mother (completed education).

O 1. Primary O 2. Secondary O 3. Pre-university

O 4. University

1. Does mother has paid work currently?

O 1. YES O 2. NO

1. How much money is the income at home monthly by all inhabitants (total income)?

O 1. More than 3000 CUP O 4. Between 500 and 1000 CUP

O 2. Between 2000 and 3000 CUP O 5. Less than 500 CUP

O 3. Between 1001 and 1999 CUP

**Prenatal history**

1. Age of mother at birth of child ______ years
2. Did mother use paracetamol during pregnancy?

O 1. Never O 2. Sometimes O 3. Frequently O 4. Daily

1. Did mother use aspirin during pregnancy?

O 1. Never O 2. Sometimes O 3. Frequently O 4. Daily

**Perinatal history**

1. Which of following options represents better color of skin of the child?

O 1. White O 2. Mixed O 3. Black

1. Weight at birth: Kilos: _____, Grams: _____

Example: if weight was 3 800 grams should write: Kilos: 3, Grams: 800

1. Height at birth:_______, ____ cm

1. How much does child weigh now? Kilos: _____, Gramos: _____

1. How much does child measure now? _______, ____ cm
2. APGAR at birth ____ / ____
3. Was the baby born for Caesarean operation?

O 1. YES O 2. NO

1. Had the child respiratory distress history at birth?

O 1. YES O 2. NO

- 1. If affirmative choose causes (mark all needed)

O 1. Hyaline membrane O 2. Meconium aspiration O 3. Other

- 1. Was mechanic ventilation used for this causes?

O 1. YES O 2. NO

- - 1. If affirmative, how many days?

O 1) 0 to 9 days O 2) 10 to 19 days O 3) 20 days or more

**Family medical history**

1. Has the child immediate family with medical diagnosis of asthma?

O 1. YES O 2. NO

- 1. If affirmative check who:

O 25.1.1 Mother O 25.1.2 Father O 25.1.3 Brothers

1. Has the child immediate family with nasal allergy (allergic rhinitis)?

O 1. YES O 2. NO

- 1. If affirmative check who:

O 26.1.1 Mother O 26.1.2 Father O 26.1.3 Brothers

1. Has the child immediate family with skin allergy (allergic dermatitis)?

O 1. YES O 2. NO

- 1. If affirmative check who:

O 27.1.1 Mother O 27.1.2 Father O 27.1.3 Brothers

**Symptoms and its characteristics**

1. Had the children wheezing, whistling, noise in chest during first year of life?

O 1. YES O 2. NO

**If answer is NO please jump to question “39”**

1. How many episodes of wheezing or whistling or noises in chest had during first year of life?

O 1. None O 2. Less than 3 episodes

O 3. 3 to 6 episodes O 4. More than 6 episodes

1. How old was baby when had the first episode of wheezing, whistling, noise in chests?

At __ __ months

1. Did episodes of wheezing, whistling or noise in chests of child were accompanied by cold?

O 1. YES O 2. NO O 3. Sometimes

1. Did the child has dry cough at night without cold or respiratory infection during first year of life?

O 1. YES O 2. NO

1. How many times have you woken up in night due to coughing with chocking, wheezing, whistling or noise in chests of the child during first year of life?

O 1. Never

O 2. Less than 1 episode per month

O 3. More than 1 episode per month or episodes that last more than a month

O 4. Continuous of permanent episodes

1. In which months during first year of life the child had wheezing, whistling or noise in chests? (you can mark more than one)

O January O February O March O April

O May O June O July O August

O September O October O November O December

1. Have been wheezing, whistling or noise in chest as severe to take child to emergency services (hospital of policlinic) during first year of life?

O 1. YES O 2. NO

1. Have been wheezing or whistling or noise in chests as severe (so strong) that you noticed him/her drowned and with difficulty for breathing during first year of life?

O 1. YES O 2. NO

1. Had the child bronchitis or bronchiolitis during first year of life?

O 1. YES O 2. NO

- 1. Had been the child hospitalized for bronchitis of bronchiolitis?

O 1. YES O 2. NO

1. Has some doctor told you that the child has asthma?

O 1. YES O 2. NO

1. Has the child had pneumonia or bronchopneumonia?

O 1. YES O 2. NO

- 1. Had been the child hospitalized for pneumonia or bronchopneumonia?

O 1. YES O 2. NO

1. How many colds has had the child during first year of life? __ __
   1. How old was the child when he/she had a cold for first time? __ __ months
2. Does the child has or have had itchy rash at the following locations: flexing sites in arms, back of knees, wrist, under the buttocks or around the neck, ears or eyes during first year of life?

O 1. YES O 2. NO

1. Does the child has or have had medical diagnosis of eczema or atopic dermatitis during first year of life?

O 1. YES O 2. NO

1. Does the child has or have had medical diagnosis of insect sting allergy during first year of life?

O 1. YES O 2. NO

1. Does the child has or have had sneezing, or white runny or stuffy nose without cold or flu during first year of life? (allergic rhinitis)

O 1. YES O 2. NO

1. Does the child has or have had treatment with inhaled medication to open bronchi (bronchodilators) by nebulizer (Salbutamol)?

O 1. YES O 2. NO O 3. DO NOT KNOW

1. Has the child received treatment with inhaled corticosteroids? (Beclomethasone, Budesonide)

O 1. YES O 2. NO O 3. DO NOT KNOW

- 1. Did symptoms relieve after treatment?

O 1. YES O 2. NO O 3. DO NOT KNOW

1. Has the child received treatment with oral or perentelar conticosteroids when he/she had wheezing, whistling of noise in chests? Example prednisone, dexametasone, prednisolone, hidrocortisone

O 1. YES O 2. NO O 3. DO NOT KNOW

- 1. Did symptoms relieve after treatment?

O 1. YES O 2. NO O 3. DO NOT KNOW

1. Has the child received treatment with oral antihistamines? Example: Loratadine, Ketotifen, other.

O 1. YES O 2. NO O 3. DO NOT KNOW

- 1. Did symptoms relieve after treatment?

O 1. YES O 2. NO O 3. DO NOT KNOW

1. Did the child received any antibiotics while when he/she had wheezing, whistling or ches noises during first year of life?

O 1. YES O 2. NO O 3. DO NOT KNOW

- 1. How many times was given antobiotics due to chest problems during first year of life?

O (1). 1 to 3 times O (3). 7 or more times

O (2). 4 to 6 times O (4). Never

1. Did the child received antibiotics for any of following causes during first year of life? (Mark with an X in right column all possible)

| - 1. Bronchitis or bronchiolitis |  |
| --- | --- |
| - 1. Cold or flu or influenza |  |
| - 1. Pneumonia or bronchopneumonia |  |
| - 1. Pharyngitis o tonsillitis |  |
| - 1. Otitis |  |
| - 1. Diarrhea |  |
| - 1. Urinary infection |  |
| - 1. Skin infection |  |
| - 1. Other causes |  |

1. Did the child received paracetamol for any reason during first year of life?

O 1. YES O 2. NO

- 1. If answered YES. How frequent did the child received treatment with paracetamol in the past 6 months?

O (1). Weekly O (3). Less than once per month

O (2). Monthly O (4). I do not remember

1. Did the child received kogrip for any reason during first year of life?

O 1. YES O 2. NO

- 1. How frequent did the child received treatment with kogrip in the past 6 months?

O (1). Weekly O (3). Less than once per month

O (2). Monthly O (4). I do not remember

1. Did the child received treatment with paracetamol or kogrip for any of the following diseases during the first year of life? (Mark an X in the right column all possible)

| - 1. Bronchitis or bronchiolitis |  |
| --- | --- |
| - 1. Cold or flu or influenza |  |
| - 1. Pneumonia or bronchopneumonia |  |
| - 1. Pharyngitis o tonsillitis |  |
| - 1. Otitis |  |
| - 1. Other cause |  |

**Lifestyle and environment**

1. Regarding technical condition of your home. How do you consider it?

O 1. Good O 2. Regular O 3. Bad

1. Regarding housing characteristics answer please:
   1. Roof: O 1. Tile O 2. Asbestos cement

O 3. Concrete (placa) O 4. Others

- 1. Walls: O 1. Wooden O 2. Mansory O 3. Others
  2. Floor: O 1. Earth O 2. Ceramic O 3 Others

1. Number of rooms of the house excluding bathroom and kitchen: _____
2. How do you consider ventilation of the house?

O 1. Good O 2. Regular O 3. Bad

1. Do you have ornamentals inside house?

O 1. YES O 2. NO

1. Has child’s house complete bathroom (sink, shower with water) inside home?

O 1. YES O 2. NO

1. Is there mold (fungi) or wet spots in the house?

O 1. YES O 2. NO

1. Kind of fuel used for cooking in the house:

O 1. Gas O 2. Coal O 3. Paraffin / kerosene O 4. Wooden O 5. Electricity O 6. Another

1. Is the kitchen of the home (place where the food if prepared) in the same room where the child sleeps?

O 1. YES O 2. NO

1. Has the child bedroom with air conditioner?

O 1. YES O 2. NO

1. Has the child curtains in bedroom or use mosquito net?

O 1. YES O 2. NO

1. Were walls in the child’s bedroom painted recently before delivery?

O 1. YES O 2. NO

- 1. If answered YES. How many months before birth? ______

1. Were walls in child’s bedroom painted recently after delivery?

O 1. YES O 2. NO

- 1. If answered YES. How many months after birth? ______

1. The crib mattress of the child is:

O 1. Of use O 2. New

1. When sleeping the child do it:

O 1. Alone O 2. Accompained (with another person)

- 1. If answered YES say by whom (you can check more than one):

O 1. Parents O 2. Brothers O 3. Grandparents O 4. Others

1. At what time was used soap to bathe the child from birth?

O 1. Before 3 months of age O 2. 3-6 months O 3. 6-12 months

O 4. After 12 months O 5. Never

1. How many times per week use soap to bathe the child?

O 1. Everyday O 2. 1-3 times O 3. 4-6 times O 4. Never

1. Is used shampoo to wash the child’s hair?

O 1. YES O 2. NO

1. Is used detergent to wash the child’s clothes (including crib clothes)?

O 1. YES O 2. NO

1. How many sibling does the child has? __ __
   1. How many of them are oldest than the child? __ __
2. How many people (adults and children) are currently living at home? __ __
3. Does the child has complete vaccines? (corresponding to the first year of life)

O 1. YES O 2. NO

1. How many hours per day the child do exercises inside of home? _____ hours.
2. How many hours per day the child do exercises outside of home? _____ hours.
3. How many hours per day the child expend watching TV? ______ hours.
4. How many months the baby was fed exclusively (only) with breastfeed (no fillers, infant formula, fruit juices or other solid foods or soups, etc)? __ __ months
5. How often the child ingest the following products (not home-made): yogurt, custard, fries packed, jellies, chocolate, fancy drinks, packed juices (soda, etc..) nectar, etc?

O 1. Never O 3. Una vez al mes

O 2. Una vez to la semana O 4.Todos los days de la semana

1. Please identify which food has been ingested by the child before:
   1. Six months (mark all possibles):

O 1. Egg yolks O 2. Beans O 3. Citrus

- 1. Nine months (mark all possibles):

O 1. Fish O 2. Smoked food or ham

- 1. 12 months of age (mark all possibles):

O 1. Whole egg O 2. Smoked food or ham

1. Aproximately how many days per week the child consume fresh vegetables? _____
2. Aproximately how many fresh vegetables consume the child per day? _____
3. Aproximately how many days per week the child consume fresh fruits? _____
4. Aproximately how many fresh fruits consume the child per day? _____
5. Did the mother smoke during pregnancy?

O 1. YES O 2. NO

1. Does the mother smoke at the moment?

O 1. YES O 2. NO

- 1. If answered YES: How many cigarettes per daily? _______

1. Does the father smoke at the moment?

O 1. YES O 2. NO

- 1. If answered YES: How many cigarettes per daily? _______

1. Do other people smoke inside the home?

O 1. YES O 2. NO

1. How many people smoke inside the home? _______
2. Do any of grandparents of the child smoke?

O 1. YES O 2. NO

- 1. If answered YES. Who?

O 1. Maternal grandfather O 2. Maternal grandmother

O 3. Paternal grandfather O 4. Paternal grandmother

1. Has the child gone to baby sitters house during first year of life?

O 1. YES O 2. NO

- 1. If answered YES: How old was the child for the first time? __ __ (months)

1. Had a pet (dog, cat, bird, hamster, rabbit) at home when the child was born?

O 1. YES O 2. NO

- 1. If answered YES mark which (mark all possible):

1. Dog.....O 2. Cat.....O 3. Others.....O

1. Have any pet at home actually (dog, cat, bird, rabbit) ?

O 1. YES O 2. NO

- 1. If answered YES mark which (mark all possible):

1. Dog.....O 2. Cat.....O 3. Others.....O

1. Is there evidence of rodents (rats, guayabitos) at home?

O 1. YES O 2. NO

1. Is there presence of vectors (cockroaches) at home?

O 1. YES O 2. NO

1. Do you think that the place where you live is a place with air pollution (fumes from factories, high traffic of vehicles, etc)?

O 1. YES O 2. NO

- 1. If answered YES please choose which (mark all possible):

O 1. Fumes or dust from factories O 2. High traffic of vehicles

- 1. If answered YES please specify the magnitude (mark one):

O 1. A lot O 2. Moderate O 3. A little

1. **Lab tests**

| **Meassures** | **Result** |
| --- | --- |
| - 1. Hemoglobin |  |
| - 1. Hematocrit |  |
| - 1. Leucogram: |  |
| - - 1. Global count |  |
| - - 1. Monocytes |  |
| - - 1. Lymphocytes |  |
| - - 1. Basophils |  |
| - - 1. Neutrophils |  |
| - - 1. Eosinophils |  |
| - 1. Total eosinophils count |  |
| - 1. Feaces |  |
